# Incidence of intraoperative hypotension and its factors among adult traumatic head injury patients in comprehensive specialized hospitals, Northwest Ethiopia: a multicenter observational study

**DOI:** 10.1186/s12871-024-02511-y

**Published:** 2024-04-01

**Authors:** Melaku Zewdu, Abraham Tarekegn Mersha, Henos Enyew Ashagre, Nurhusen Riskey Arefayne, Biresaw Ayen Tegegne

**Affiliations:** 1 Department of Anesthesia, School of Medicine, College of Medicine and Health Sciences, Debre Tabor University, Debre Tabor, Ethiopia; 2 Department of Anesthesia, School of Medicine, College of Medicine and Health Sciences, University of Gondar, Gondar, Ethiopia

**Keywords:** Traumatic head injury, Intra-operative hypotension, Outcome

## Abstract

**Introduction:**

Traumatic head injury (THI) poses a significant global public health burden, often contributing to mortality and disability. Intraoperative hypotension (IH) during emergency neurosurgery for THI can adversely affect perioperative outcomes, and understanding associated risk factors is essential for prevention.

**Method:**

A multi-center observational study was conducted from February 10 to June 30, 2022. A simple random sampling technique was used to select the study participants. Patient data were analyzed using bivariate and multivariate logistic regression to identify significant factors associated with intraoperative hypotension (IH). Odds ratios with 95% confidence intervals were used to show the strength of association, and *P* value < 0.05 was considered as statistically significant.

**Result:**

The incidence of intra-operative hypotension was 46.41% with 95%CI (39.2,53.6). The factors were duration of anesthesia ≥ 135 min with AOR: 4.25, 95% CI (1.004,17.98), severe GCS score with AOR: 7.23, 95% CI (1.098,47.67), intracranial hematoma size ≥ 15 mm with AOR: 7.69, 95% CI (1.18,50.05), and no pupillary abnormality with AOR: 0.061, 95% CI (0.005,0.732).

**Conclusion and recommendation:**

The incidence of intraoperative hypotension was considerably high. The duration of anesthesia, GCS score, hematoma size, and pupillary abnormalities were associated. The high incidence of IH underscores the need for careful preoperative neurological assessment, utilizing CT findings, vigilance for IH in patients at risk, and proactive management of IH during surgery. Further research should investigate specific mitigation strategies.

## Introduction

Traumatic head injury (THI) poses a significant global public health burden, often contributing to mortality and disability [1]. Approximately 1.7 million people sustain THI every year in the United States, and which contributed approximately 30.5% of all injury-related deaths [2]. Its prevalence contributes about 41% emergency cases in sub-Saharan African countries [3]. It has estimated about 40.5% of all traumatic related admissions in Ethiopia [4, 5]. The problem mostly affects the working age group between 17 and 49 years, and the indications for surgery are compound depressed skull fracture, intracerebral hematoma, subdural hematoma, extradural hematoma, and impacted intracranial foreign bodies [6].

Despite modern diagnosis and clinical management, patients with THI have poor prognosis [7]. Although the severity of primary injury is a major factor that predicts mortality and morbidity, secondary injury caused by physiological insults, such as intraoperative hypotension(IH), can also worsen the outcome [1, 7]. In the course of THI management, surgery and anesthesia may contribute to the onset of hypotensive insults, which can lead to adverse clinical outcomes in approximately 60% [8]. Although there are a variety of definitions for IH, the most commonly used is an SBP less than 90 mmHg during the surgical procedure [9]. Approximately 24% to 62.9% of patients who undergo emergency craniotomy have developed intra operative hypotension [10]. Hypotension after intubation is associated with increased mortality due to acute kidney injury, bowel ischemia, cardiac ischemia, and increased brain insult [11]. The insult is even worse in patients with head injury [10, 12]. During surgical procedure intractable hypotension, which is unresponsive to vasopressors, often occurs after dural opening and leads to cardiac arrest [7]. Patients who developed intraoperative hypotension was 32% having three times post-operative mortality rate than who remained normotensive throughout surgery [13].

Female sex, systolic blood pressure before general anesthesia, and American Society of Anesthesiology(ASA) status were factors for the development of IH [14]. Moreover, multiple CT lesions, subdural hematoma, and a longer duration of anesthesia are associated with IH [15]. A low GCS score (< 5), tachycardia, preoperative hypertension, and delayed surgery > 3 h until surgery were strongly associated with IH [9]. Avoiding vasopressor or fluid administration for patients undergoing neurosurgery may expose to unnecessary episodes of intraoperative hypotension [9, 16]. Delayed surgery, anesthesia duration, opioid administration, patient age, and documented medical history of ischemic heart disease, diabetes mellitus, and hypertension were also associated with IH [15, 17]. The studies have found that reducing the incidence of intraoperative hypotension lowers the chances of oxidative stress and organ damage [18–20]. Intraoperative hypotension (IH) during emergency neurosurgery for THI can adversely affect perioperative outcomes, and understanding associated risk factors is essential for prevention. Therefore, this study aimed to assess the incidence of intraoperative hypotension and its risk factors in patients with traumatic head injuries.

## Methods

### Study design, study area and period

A multicenter observational study was conducted from February 10 to June 30, 2022, at comprehensive specialized hospitals in the Amhara regional state, North west Ethiopia: Debre Tabor, Felege Hiwot, Tibebe Gion, and University of Gondar Comprehensive specialized hospitals. These hospitals serve more than eight million populations in each center and are the final referral choices for other health institutions that provide tertiary-level emergency care. Each hospital had more than 33 specialists and an average of more than six operation room theaters.

### Source population

All adult patients with traumatic head injuries underwent emergency surgery in the Amhara regional state, North west Ethiopia: Debre Tabor, Felege Hiwot, Tibebe Gion, and University of Gondar Comprehensive specialized hospitals.

### Study population

All adult patients with traumatic head injuries underwent emergency surgery in the Amhara regional state, North west Ethiopia: Debre Tabor, Felege Hiwot, Tibebe Gion, and University of Gondar Comprehensive specialized hospitals until the calculated sample size was reached.

### Eligibility criteria

#### Inclusion criteria

Patients aged ≥ 18 years who were brought to the operating room directly from the emergency department for traumatic head injury surgery in the Amhara regional state, North west Ethiopia: Debre Tabor, Felege Hiwot, Tibebe Gion, and University of Gondar Comprehensive specialized hospitals were included.

#### Exclusion criteria

Patients who underwent repeat craniotomy, non-emergent craniotomy, burr hole evacuation under local anesthesia, raised intracranial pressure (ICP), polytrauma with shock were excluded from the study.

### Sample size determination and sampling procedure

#### Sample size determination

The sample size was calculated using a single-population proportion formula. We have used a 95% confidence level, margin of error (0.05), and assumed maximum variability (*p* = 0.5). These parameters are substituted into the following single-population proportion formula:

$${\text{n}}=\frac{{{\text{z}}}^{2}{\text{p}}\left(1-{\text{p}}\right)}{{{\text{d}}}^{2}}=\frac{{\left(1.96\right)}^{2}\times 0.5{\left(1-0.5\right)}^{2}}{{0.05}^{2}}=384$$

where, *n* = Sample size

*p* = Proportion of people

d = margin of error (0.05)z = confidence level 95% (i.e. 1.96)

However, according to the information obtained from postoperative surgical note documentation, the average number of surgeries for traumatic head injury was approximately 8 at Debretabor,16 at the university of Gondar, 19 at Tibebe Gion, and 14 at Felege Hiwot comprehensive specialized hospitals, making a total of 57 emergency surgeries per month and about 684 annually. Since the total population is less than 10,000, we used the reduction formula, and when this value (684) is evenly distributed in five months on average of 285 patients undergoing emergency surgery for traumatic head injury.

Therefore, the sample size was calculated as Nf = n_o_/1 + n_o_/N, 384/1 + 384/285 = 164.

When 10% nonresponse rate was used, the final sample size was about 181.

Where **nf**: final sample size.

**n**_**o**_: sample size from the single proportion formula and.

**N**: average number of populations on selected hospitals per study period.

Approximately 51 patients from the University of Gondar, 60 from Tibebe Gion, 45 from Felege Hiwot, and 25 from Debretabor Comprehensive Specialized Hospitals were used.

#### Sampling procedure

Computer generated simple random sampling teqnique was used to select study participants.

### Study variables

#### Dependent variable

Intraoperative hypotension was observed every five minutes on the monitor throughout the surgery and anesthesia time.

#### Independent variables

*Personal and clinical variables* included sex, age, body mass index, comorbidities (hypertension, diabetes mellitus, and cardiac illness), baseline vital signs, and GCS scores.

*Surgical operation related variables* the type of operation (elevation, craniotomy, burr hole), total blood loss, CT findings (mid line shift and hematoma size), duration of the operation, and delay of surgery.

*Anesthesia and medication related variables:* ASA status, opioid use, use of vasoactive agents, and duration of anesthesia.

### Operational definitions

*Traumatic Head Injury (THI):* is a vast array of injuries that occur on the scalp, skull, brain, underlying tissues, and blood vessels of the head.

*Intraoperative hypotension:* SBP < 90 mmHg (irrespective of the duration of the episode) [9, 21–23].

*Bradycardia:* defined as heart rate < 60 beats per minute.

*Tachycardia:* defined as heart rate > 100 beats per minute.

*Maximum thickness of CT lesion* was defined as the largest dimension of the hemorrhagic lesion reported on an immediate preoperative CT scan by a clinical radiologist.

*Glasgow Coma Scale (GCS):* classifies traumatic brain injuries as mild [14, 15]; moderate [9–13] and severe [3–8].

*Body mass index (BMI):* classifies the weight status as underweight (below 18.5), normal (18.5–24.9), overweight (25–29.9), and obesity (30 and above).

### Data collection tools and procedure

Informed consent was obtained from each participant or immediate surrogate (not cooperative due to traumatic injury), and data were collected using a semi-structured questionnaire based on the literature review of previous studies. It has been modified and used in the present study [15, 17, 24–26].

### Data quality control

Two data collectors and one supervisor at each institution were trained for each item included in the study tool: objective, relevance of study, and rights of respondents. Regular supervision and follow-up were performed during the data collection. The investigator cross-checked the completeness and consistency of the data on a weekly basis.

### Data processing and analysis

After data collection, the data were entered into Epi data (V4.6.0.4) computer software and transported to IBM SPSS version 20 for analysis. Descriptive statistics were used to show baseline clinical characteristics and IH. To examine the differences in variables between patients with and without IH, the chi-squared test was used. Binary logistic regression was used to determine the association between each independent variable and the outcome variable. Multivariable logistic regression analysis was used to determine independent risk factors for IH. Data are reported as median ± IQR), in percentage, and odds ratio (OR) with 95% confidence intervals (CI). Statistical significance was set at *p* < 0.05.

## Result

### Personal and clinical characteristics

Among study participants, 163(90.06%) of them were male with median age of 31 (23,45) year. There were only 3.31% of patients with comorbidities who underwent emergency neuro surgical procedures, and 2.76% of them had developed IH. Only 14.36% of patients have had a baseline heart rate of (HR) ≥ 112, of whom 9.94% had developed IH. A baseline systolic blood pressure (SBP) of ≥ 140 mmHg was observed in approximately 19.89%, with 7.74% of them developing IH. Among 22.7% of patients who presented with severe GCS scores, IH has occurred in approximately 18.82% (Table 1).

**Table 1:** Cross-tabulation of personal and clinical characteristics in relation to intraoperative hypotension, North-west Ethiopia, *February 10 to June 30, 2022*

		Observations	Percent	Intraoperative hypotension	
				Yes (N&%)	No (N&%)
Sex	Male	163	90.06	74(40.89)	89(49.17)
	Female	18	9.94	10(5.52)	8(4.42)
BMI	Under weight	5	2.78	1(.56)	4(2.22)
	Normal	168	92.81	78(43.09)	90(49.72)
	Overweight	6	3.31	4(2.21)	2(1.1)
	Obese	2	1.10	1(.55)	1(.55)
Co-morbid disease	Yes	6	3.31	5(2.76)	1(.55)
	No	175	96.69	79(43.65)	96(53.04)
Baseline HR	HR ≥ 112	26	14.36	18(9.94)	8(4.42)
	HR < 112	155	85.64	66(36.47)	89(49.17)
Baseline SBP (mmHg)	SBP ≥ 140	36	19.89	14(7.74)	22(12.15)
	SBP < 140	145	80.11	70(38.67)	75(41.44)
GCS level	Mild	92	50.8	25(13.80)	67(37.00)
	Moderate	48	26.5	25(13.80)	23(12.70)
	Sever	41	22.7	34(18.82)	7(3.88)
	Median		25th percentile (IQR)		75th percentile (IQR)
Age(year)	31		24		45

*GCS* Glasgow Coma Scale, *SBP* systolic blood pressure, *mmHg* millimeter mercury, *HR* heart rate, *BMI* body mass index, *IQR* interquartile range

In this study, the incidence of intraoperative hypotension (IH) was approximately 46.41%, with 95%CI (39.2%, 53.6%). The majority 60(33.15%) of patients were from University of Gondar Comprehensive Specialized Hospital, and 14.92% of them had developed IH (Table 2).

**Table 2:** Distribution of intraoperative hypotension respective to each study center, North west Ethiopia, *February 10 to June 30, 2022*

Study center (comprehensive specialized hospitals)	Intraoperative hypotension		Total
	Yes (N&%)	No (N&%)	
UoG Hospital	27(14.92)	24(13.26)	51
Tibebe Gion Hospital	29(16.02)	31(17.13)	60
Felege Hiwot Hospital	20(11.05)	25(13.81)	45
Debre Tabor Hospital	8(4.42)	17(9.39)	25
Total	84(46.41)	97(53.59)	181

*UoG* University of Gondar

### Anesthesia and medication related factors

The majority 148(81.77%) of patients were ASA I-III, and 56(30.94%) of them had developed intraoperative hypotension. Around 2/3^red^ 118(65.19%) of emergency neurosurgical procedures were performed with the induction agent of thiopentone, and IH was occurred in approximately 28.18% of theme. There was a prolonged duration of anesthesia (≥ 135 min) in 51(28.18%) of patients, and intra-operative hypotension was observed in 19.34%. Approximately 80% of the patients were used opioids as analgesics of choice, and 36.47% of them were developed IH. Approximately 4.42% of the patients have received adrenalin where as 17.68% of the patients received used mannitol intraoperatively (Table 3).

**Table 3:** Cross-tabulation of anesthesia and medication related factors with intraoperative hypotension, North West Ethiopia, *February 10 to June 30, 2022*

		Observations	Percentile	Intraoperative hypotension	
				Yes (N&%)	No (N&%)
ASA status	ASA > III	33	18.23	28(15.47)	5 (2.7)
	ASAI-III	148	81.77	56(30.94)	92 (50.83)
Induction agent	Thiopentone	118	65.19	51(28.18)	67(37.01)
	Ketamine	23	12.71	13(7.18)	10(5.53)
	Propofol	28	15.47	16(8.84)	12(6.63)
	Ketofol	12	6.63	4(2.21)	8(4.42)
Maintenance agents	Isoflurane	157	86.74	74(40.88)	83(45.86)
	Halothane	24	13.26	10(5.53)	14(7.73)
Duration of anesthesia	≥ 135 min	51	28.18	35(19.34)	16(8.84)
	< 135	130	71.82	49(27.07)	81(44.75)
Opioids	Yes	144	79.56	66(36.47)	78(43.09)
	No	37	20.44	18(9.94)	19(10.50)
Adrenaline	Yes	8	4.42	7(3.87)	1(.55)
	No	173	95.58	77(42.54)	96(53.04)
Mannitol	Yes	32	17.68	17(9.39)	15(8.29)
	No	149	82.32	67(37.02)	82(45.30)
	Median	25th percentile		75th percentile	
Intra-op fluid	2000 ml	1700 ml		2300 ml	
Total blood loss	200 ml	400 ml		675 ml	

Ketofol is a 1:1proportion combined dose of ketamine and propofol

*ASA* American Society of Anesthesiologists, *ml* milliliter

### Surgical procedure related factors

Approximately half 84(48.07%) of the patients underwent elevation, and 17.13% of theme were developed IH. More than 2/3rd 126(69.6%) of the patients were performed surgery after 8 h after the incident, and 32.04% of them had developed IH. Among 49(27.07%) of patients who had a midline shift in the CT-scan, IH has occurred in approximately 20%. More than 2/3rd 126(69.61) of the patients had hematoma size ≥ 15 mm, and 32.60% had developed IH (Table 4).

**Table 4:** Cross tabulation of surgical procedure related factors with intraoperative hypotension, North West Ethiopia, *February 10 to June 30, 2022*

		Observations	Percent	Intraoperative hypotension	
				Yes (N&%)	No (N&%)
Type procedure	Elevation	87	48.07	31(17.13)	56(30.94)
	Craniotomy	77	42.54	48(26.52)	29(16.02)
	Bar hole	17	9.39	4(2.21)	13(7.18)
Time to surgical operation	Within 8 hr	55	30.4	26(14.37)	29(16.03)
	> 8 hr	126	69.6	58(32.04)	68(37.56)
CT scan midline shift	Yes	49	27.07	35(19.34)	14(7.73)
	No	132	72.93	51(28.18)	81(44.75)
Hematoma size	≥ 15 mm	126	69.61	59(32.60)	67(37.01)
	< 15 mm	55	30.39	14(7.74)	41(22.65)
Pupillary abnormality	Abnormal pupil	22	12.15	16(8.84)	6(3.31)
	Normal pupil	157	87.85	66(36.93)	91(50.92)

*CT* computed tomography, *mm* millimeter

### Bivariable and multivariable analysis for factors associated with intraoperative hypotension

Bivariable logistic regression showed that ASA status, baseline HR, GCS score, duration of anesthesia, type of procedure, intra-operative blood loss, total intraoperative fluid, CT-scan midline shift, hematoma size, and pupillary abnormality were associated with intraoperative hypotension (*p* < 0.25).

Patients who had ≥ 135 min duration of anesthesia, severe GCS score, hematoma size of greater than 15 mm, and abnormal pupil size were significantly associated with intraoperative hypotension in multivariable logistic regression at a *p*-value of < 0.05.

Patients who have ≥ 135minut duration of anesthesia were approximately four times more likely to have intraoperative hypotension than < 135 min’ anesthesia duration [AOR: 4.25, 95% CI (1.004,17.98)]. Patients who have severe GCS scores were approximately seven times more likely to have intraoperative hypotension than those without [AOR, 7.23; 95% CI (1.098,47.67)]. The odds of intraoperative hypotension were approximately 7.7 times higher in patients with hematoma size of greater than 15 mm compared to those hematoma size of less than 15 mm [AOR: 7.69, 95% CI (1.18–50.05)]. Patients with normal pupillary size had decreased the odds of occurrence of intra operative hypotension by 94% compared to patients whose pupil was abnormal [AOR, 0.061; 95% CI (0.005—0.732)] (Table 5).

**Table 5:** Bivariable and multivariable logistic regression analysis of factors associated with intraoperative hypotension, North West Ethiopia, *February 10 to June 30, 2022*

Overall variables (181)		IOP hypotension				
		Yes (N&%)	No (N&%)	Odds- ratio		
		84(46.4%)	97(53.6%)	Cruds (95%CI)	Adjusted (95%CI)	*P*-value
Baseline HR	HR ≥ 112	18(69.23)	8(30.07)	3.0(1.23, 7.32)	2.44 (0.21, 27.98)	0.473
	HR < 112	66(42.58)	89(57.41)	1	1	1
GCS level	Sever	34(82.92)	7(17.07)	13.02(5.11, 33.13)	7.23(1.1, 47.67)	0.04
	Moderate	25(52.08)	23(47.91)	2.91(1.41, 6.04)	1.147(0.26,5.03)	0.855
	Mild	25(27.17)	67(72.82)	1	1	
ASA status	ASA > III	28(84.84)	5 (15.15)	9.2(3.36, 25.26)	0.743(0.12,4.46)	0.745
	ASAI-III	56(37.83)	92 (62.16)	1	1	
Duration of anesthesia	≥ 135 min	35(68.62)	16(31.37)	4.18(2.10, 8.31)	4.25(1.004,17.98)	0.049
	< 135 min	49(37.69)	81(62.30)	1	1	
IV fluid	Fluid used			1.001(1.001,1.002)	1.001(1.00,1.003)	0.115
Type procedure	Elevation	30(35.7)	54(64.28)	2.41(0.64, 9.12)	1.48(0.08,25.06)	0.788
	Craniotomy	49(66.21)	29(39.1)	7.32(1.92, 27.87)	9.68(0.89, 88.95)	0.063
	Bar hole	3(18.75)	13(81.25)	1	1	
IOP blood loss	≥ 10 ml/kg	32(17.68)	6(3.32)	1.001(1.00,1.001)	1.0(0.998, 1.001)	0.672
	< 10 ml/kg)	53(29.28)	90(49.72)			
CT scan midline shift	Yes	30(76.92)	9(23.07)	5.51(2.40, 12.63)	4.19(0.94, 18.61)	0.06
	No	46(37.70)	76(62.79)	1	1	
Hematoma size	≥ 15 mm	36(57.14)	27(42.85)	4.0(1.49, 10.77)	7.7(1.18, 50.04)	0.033
	< 15 mm	7(25)	21(75)	1	1	
Pupillary abnormality	No	66(42.03)	91(57.96)	0.27(0.10,0.73)	0.061(0.005,0.73)	0.027
	Yes	16(72.72)	6(27.27)	1	1	

*GCS* Glasgow Coma Scale, *IV* intravenous, *HR* heart rate, *BMI* body mass index, *ASA* American Society of Anesthesiologists, *CI* confidence interval, *CT* computed tomography, *IOP* intraoperative, *mm* millimeter

## Discussion

This study aimed to determine the incidence of IH and identify personal, anesthetic, and surgical procedure-related factors for the occurrence of intraoperative hypotension among adult emergency neurosurgical patients.

In this study, the overall incidence of intraoperative hypotension among the total observations was 46.4%. This study is in line with the studies conducted in children and adult around 52%and 54% respectively [27, 28]. However, studies in New York and Japan have shown low incidence of intraoperative hypotension which is approximately 27.9% and 32%, respectively) [7, 13]. A possible reason for the variation in the current study could be the difference in sample size. In contrast, a study conducted at University of Washington and Tokyo have found higher occurrence of intraoperative hypotension, accounting 65% and 67% respectively. Thus, the higher results from the current study may be due to differences in the observations regarding IH occurrence [15, 26]. The current study revealed that IH was high in patients diagnosed with cerebral contusions, subdural hematoma, or epidural hematoma during emergency neurosurgery. A possible reason might be an increased preoperative ICP, which is susceptible to intraoperative reduction in blood pressure due to surgery and anesthesia [10, 29].

The current study showed that the duration of anesthesia (≥ 135 min) was a factor for IH, which is supported by a study conducted at the University of Washington [15]. A possible reason for this association in the current study could be the commonly used anesthetic agents that might result in myocardial depression and peripheral vasodilation, leading to intraoperative hypotension as the duration of exposure increases. In this study, a severe GCS score was associated with IH incidence, which is consistent with a study conducted in China [10]. A study conducted in Thailand revealed that severe GCS scores increased the occurrence of intraoperative hypotension four times [30]. The findings of the current study are supported by a study conducted in Japan [9]. However, another study reported that GCS score was not a predictor of IH occurrence [7]. This variation could be attributed to the definition used for intraoperative hypotension and measurement. A possible reason for the association between severe GCS score and IH is that a poor GCS score might be associated with the risk of brain hemorrhage and concomitant increase in intracranial pressure; therefore, following the surgery, the sympathetic tone might be lost and IH occurs [31]. Moreover, patients with low GCS scores may be at a high risk of hypoxia and brain swelling due to respiratory depression or apneic episodes, resulting in hemodynamic instability.

In this study, hematoma size ≥ 15 mm was a factor of intraoperative hypotension, which is supported by a study conducted in Nippon Tokyo [26]. The reason behind this is that blood in the cranial vault reflects depletion of the same amount of blood from the systemic circulation, and a sudden decrease in sympathetic tone during surgical evacuation may result in vasodilatation and hypotension [32–35]. Pupillary abnormalities have been associated with intraoperative hypotension, consistent with the other study [33]. This association can be explained by pupil reactivity being a sensitive measure and an early indicator of an increase in ICP, which mostly occurs because of cranial nerve compression [36]. According to a study conducted in California, patients with head injuries experienced unexpected hypotension resulting from mannitol administration [37]. However, the current study found no significant association with the use of diuretics (mannitol), which is consistent with the findings of other studies [10, 15]. A possible reason for this variation may be the difference in dose adjustment and infusion rates intraoperatively.

Studies have found an association between a high preoperative blood pressure with IH [9, 28]. However, the current study found no association between the preoperative blood pressure with IH. This variation might be due to the differences in the response of clinicians to IH stabilization. In this study, total fluid consumption was not significantly associated with IH. This may be due to the controversy regarding the deleterious effects of both large and restricted crystalloid volumes on intracranial pressure following traumatic head injury [9]. However, there has been increased use of small-volume resuscitation with hypertonic saline and dextran to restore hemodynamic stability after traumatic brain injury [38]. Furthermore, in the perioperative period, employing a therapeutic approach that adapts fluid resuscitation based on the identification of preload responsiveness, along with careful fluid status assessment and hemodynamic monitoring, reduces postoperative complications [39, 40].

This study found an association between high-grade ASA (> 3) and IH [22]. This possible variation could be due to differences in the study population. However, the current study found no association between high-grade ASA (> 3) and IH, which is supported by a study conducted in Japan [14]. A study has found an association between midline shift of brain CT and IH, since this may indicate higher intracranial pressure that can result in sudden loss of sympathetic tone, leading to hypotension [15]. In contrast, the current study found no association between midline shift in brain CT and IH, which is in line with a study conducted in Thailand [28]. This variation may be due to differences in sample size [15].

### Strength and limitation

As far as our search concerned, we cannot find similar studies in Ethiopia. Therefore, this may help as a baseline data for other studies. This multicenter study can provide new insights into minimizing intraoperative hypotension in patients with traumatic head injuries.

However, we depended on reading non-invasive blood pressure every five minute for the occurrence of hypotension, and it would be very accurate if continuous intra-arterial blood pressure measurement was used, which may precisely report the duration or timing of hypotension.

## Conclusion and recommendation

The incidence of intraoperative hypotension was considerably high. The duration of anesthesia, GCS score, hematoma size, and pupillary abnormalities were associated. The high incidence of IH underscores the need for careful preoperative neurological assessment, utilizing CT findings, vigilance for IH in patients at risk, and proactive management of IH during surgery. Further research should investigate specific mitigation strategies.

## Data Availability

Data for this study is available on reasonable request from corresponding author.

## Abbreviations

ASA: American Society of Anesthesiologists
BMI: Body Mass Index
BP: Blood Pressure
GCS: Glasgow Coma Scale
IH: Intraoperative Hypotension
ICP: Intracranial Pressure
MAP: Mean Arterial Pressure
SBP: Systolic Blood Pressure
TBI: Traumatic Brain Injury
THI: Traumatic Head Injury
UOGCSH: University of Gondar Comprehensive Specialized Hospital
